# Expanded Dengue and the Digestive System: A Systematic Review and Meta-Analysis

**DOI:** 10.3390/tropicalmed11030077

**Published:** 2026-03-07

**Authors:** Daniel Peñaherrera-Vásquez, Alison Reina, Gabriela Zambrano-Sánchez, Maria Fernanda García-Aguilera, German Fierro, Silvia Jessica Guarderas-Muñoz, Josue Rivadeneira, Luis Fuenmayor-González

**Affiliations:** 1 Unidad de Revisiones Sistemáticas y Metaanálisis-URMA, Facultad de Ciencias Médicas, Universidad Central del Ecuador, Quito 170403, Ecuador; eliteitor@hotmail.com (D.P.-V.); dra.gzambrano@gmail.com (G.Z.-S.); mafergarcia.g@gmail.com (M.F.G.-A.); zk2300@hotmail.com (G.F.); sjguarderas@uce.edu.ec (S.J.G.-M.); 2 Zero Biomedical Research, Quito 170103, Ecuador; fluis@wustl.edu; 3 Carrera de Laboratorio Clínico, Facultad de Ciencias de la Salud, Universidad Técnica de Ambato, Ambato 180207, Ecuador; gabali4-flores@hotmail.com; 4 Hospital de Especialidades Eugenio Espejo, Quito 170403, Ecuador; 5 Doctorado en Ciencias Médicas, Universidad de La Frontera, Temuco 01145, Chile; 6 School of Public Health, Washington University in St. Louis, St. Louis, MO 63130, USA

**Keywords:** dengue, gastrointestinal diseases, dengue/complications, atypical symptoms, expanded dengue, systematic reviews, meta-analysis

## Abstract

**Background** Expanded dengue syndrome represents a severe and atypical spectrum of dengue virus infection characterized by multisystem involvement beyond classic manifestations. While mild gastrointestinal symptoms are common in classic dengue, expanded dengue syndrome may present with clinically significant digestive organ involvement, including hepatitis, fulminant hepatic failure, pancreatitis, and acute acalculous cholecystitis. These manifestations often resemble primary gastrointestinal diseases, leading to diagnostic delays and inappropriate management. Despite increasing recognition, the true frequency of digestive system involvement remains poorly defined due to heterogeneous reporting and limited quantitative evidence. **Methodology/Principal Findings** A systematic review and meta-analysis was conducted in accordance with the Preferred Reporting Items for Systematic Reviews and Meta-Analyses guidelines and registered in the International Prospective Register of Systematic Reviews (PROSPERO; CRD420251270772). MEDLINE, Scopus, Web of Science, Embase, CENTRAL, Scielo, and BIREME were searched from inception to December 10, 2025. Primary studies reporting laboratory-confirmed dengue infection with atypical digestive system involvement and sufficient quantitative data were included. Seven studies comprising 1774 participants met eligibility criteria. Random-effects meta-analyses were performed to estimate pooled frequencies of gastrointestinal manifestations. The pooled frequency of hepatic involvement was 7% (95% confidence interval: 0–21), including fulminant hepatic failure (3%) and hepatitis (33%), with substantial heterogeneity. Acute pancreatitis occurred in 3% (95% confidence interval: 0–11) of cases. Acute acalculous cholecystitis was the most frequent manifestation, with a pooled frequency of 21% (95% confidence interval: 3–48). All included studies were classified as low risk of bias.

## 1. Introduction

Expanded dengue refers to a clinical form of dengue virus infection characterized by a wide spectrum of atypical manifestations that extend beyond the classic features of dengue fever, such as fever, retro-orbital pain, and rash. It encompasses confirmed dengue infections with multisystem involvement, including neurological, cardiovascular, hepatic, renal, gastrointestinal, pulmonary, and hematological manifestations with or without plasma leakage. These presentations do not fully meet the conventional World Health Organization criteria for dengue haemorrhagic fever or dengue shock syndrome, yet are recognized as part of the severe clinical spectrum of dengue. Expanded dengue is frequently associated with diagnostic challenges, poor response to standard management, and an increased risk of severe complications and mortality [1,2,3].

Expanded dengue is more prevalent in endemic dengue areas, particularly in tropical and subtropical regions where the dengue virus is present, such as Southeast Asia, Latin America, and Africa. The prevalence of expanded dengue is difficult to determine due to the diversity of its clinical manifestations and the lack of a strict definition in the medical literature [4].

Classic dengue is generally associated with mild digestive symptoms, such as abdominal pain, nausea, or vomiting, which are common during the febrile phase of the illness. There may also be manifestations such as splenomegaly, hepatomegaly, and, in some cases, alterations in liver function tests. These symptoms are typically part of the body’s inflammatory response to the virus, without causing severe damage to the digestive organs [5].

However, in expanded dengue, the digestive system can become a central focus of the disease. In addition to common gastrointestinal symptoms, expanded dengue can cause more severe damage to the liver and other organs within the digestive system. Cases of viral hepatitis have been documented, with significant alterations in liver enzymes, and liver failure in the most severe cases. Additionally, patients may present with pancreatitis, cholecystitis, and appendicitis, further complicating the course of the diseases [6].

It is crucial to recognize digestive manifestations in the context of expanded dengue, as their presentation is often confused with other gastrointestinal pathologies, delaying an early diagnosis that is essential to prevent complications such as multi-organ failure. For healthcare professionals, understanding this involvement is of vital importance to identify critical cases early and provide targeted treatment. However, the lack of consensus on its true incidence highlights the imperative need for a meta-analysis to scientifically determine the frequency of expanded dengue within the digestive system. This consolidation of evidence is essential to standardize clinical criteria and improve the prognosis of a condition where, currently, no study conclusively establishes an exact frequency.

## 2. Methods

This manuscript has been prepared in accordance with PRISMA guidelines (Preferred Reporting Items for Systematic Reviews and Meta-Analyses) [7].

### 2.1. Study Protocol

Registered in PROSPERO (International Prospective Register of Systematic Reviews, NIHR), ID: CRD420251270772

### 2.2. Study Design

Systematic review.

### 2.3. Eligibility Criteria

Primary studies assessing patients of any age with atypical digestive system involvement in the context of Expanded Dengue Syndrome (EDS) were included. Atypical digestive involvement was defined as clinically relevant alterations affecting one or more of the following organs: liver, pancreas, esophagus, stomach, small intestine, large intestine, rectum, or gallbladder [1,2,3]. Studies reporting exclusively typical gastrointestinal manifestations of dengue were excluded. Typical digestive manifestations were defined as nausea, vomiting, abdominal pain, anorexia, diarrhea, epigastric discomfort, mild hepatomegaly, and mild elevations of liver enzymes without evidence of clinically significant organ dysfunction [4]

Eligible studies were required to include laboratory-confirmed dengue infection, defined by reverse transcription polymerase chain reaction (RT-PCR), NS1 antigen detection, viral isolation, or serological confirmation (IgM and/or IgG), according to the diagnostic criteria applied in each study.

No language restrictions were considered. Eligible studies included analytical observational designs (cohort, case–control, and cross-sectional studies), as well as randomized and non-randomized clinical trials. Only studies providing sufficient quantitative data to allow valid estimation of the frequency of atypical gastrointestinal manifestations among patients with EDS, specifically reporting both a numerator (number of patients with atypical digestive involvement) and an appropriate denominator (total number of patients with EDS), were included.

Case reports, case series, and conference abstracts without primary data were excluded. In addition, letters to the editor, expert opinions, commentaries, editorials, narrative reviews, and other non-original studies were excluded. Studies conducted in hospital and non-hospital settings involving patients with Expanded Dengue Syndrome were eligible for inclusion.

### 2.4. Information Sources

The databases consulted included MEDLINE via PubMed, Scopus, Web of Science, Embase, CENTRAL (Cochrane Central Register of Controlled Trials), Scielo, and BIREME-Biblioteca Virtual de Salud from their inception until December (the last search was conducted on 10 December 2025). Search terms included adapted combinations of “Expanded Dengue,” “gastrointestinal disease”, and “frequency,” using Boolean operators and field tags were available.

### 2.5. Search Strategies

This was conducted using the PECO framework (Population [P: patients with gastrointestinal symptoms or diseases], Exposure [E: Expanded Dengue Syndrome], Comparator [C: none], and Outcome [O: frequency]). Sensitive searches were performed by adapting the strategy to each information source, using MeSH, DeCS, and Emtree terms, as well as free-text terms. The search strategy was tailored to each database (Supplementary File S1).

In addition to database searches, we conducted a targeted screening of reference lists. During full-text review, when an included article cited another study that appeared potentially relevant based on its title or context, we retrieved the cited article and evaluated it against our eligibility criteria. We repeated this process iteratively until no additional eligible studies were identified.

### 2.6. Selection Process

The documents identified from each information source were organized with Rayyan Software (Rayyan Systems Inc., Cambridge, MA, USA). Duplicates were eliminated through both automated and manual methods. Next, two pairs of reviewers (AR and DP-V) independently and blindly screened the titles and abstracts. Any disagreements were resolved through consensus. The manuscript recruitment process was finished on 18 December 2025.

### 2.7. Data Collection and Analysis

A Microsoft Excel spreadsheet (PC Excel, version 15.24; 2016 Microsoft Corporation) was developed for data extraction. Two authors (GZ and MFG-A) extracted data from the included studies, while three others (LF-G, SJG-M, JR) verified the extracted data. Any discrepancies between reviewers were resolved through consensus.

### 2.8. Variables

The variables analyzed included publication year, geographic location (country), population size, number of cases, mean age, sex, study design, and setting. We also retrieved the gastrointestinal condition described, the population age group (pediatric, adult, or both), and the diagnostic tool used for dengue diagnosis. Tables provide a narrative summary of the general characteristics of all included studies. Given the topic’s relevance and the need to add value by quantifying the frequency of gastrointestinal involvement associated with EDS, a meta-analysis was performed using a random-effects model and a restricted maximum-likelihood method.

### 2.9. Individual Biases and Quality

Risk of bias for individual studies was assessed using the JBI critical appraisal checklist for frequency studies [8,9,10]. This instrument evaluates nine aspects: (D1) adequacy of the sampling frame, (D2) recruitment method, (D3) sample size, (D4) study subjects and setting, (D5) coverage, (D6) diagnostic methods, (D7) reliability and standardization of measurements, (D8) statistical analysis, and (D9) response rate. Two reviewers (DP-V and AR) independently evaluated each item, marking it as “yes,” “no,” “unclear,” or “not applicable.” Discrepancies were discussed and resolved by consensus. The percentage of affirmative responses determined overall RoB, classifying studies as “high” risk (≤49%), “moderate” risk (50–69%), or “low” risk (≥70%). RoB summary plots across domains were generated using the ROBVIS R package v.0.3.0.90013.

### 2.10. Synthesis Method

A narrative synthesis of the results is presented in tables. Three meta-analyses were performed using studies reporting liver, pancreatic, and gallbladder involvement, respectively. To stabilize inter-study variance (heterogeneity), the Freeman-Tukey double arcsine transformation was applied to the proportions data. Statistical heterogeneity among the included studies was quantified using Cochran’s Q test and the I^2^ statistic. The Q test was considered statistically significant at a *p*-value <0.05. Additionally, univariate meta-regression analyses were conducted to formally investigate potential sources of the observed heterogeneity, using the following prespecified covariates: sample size, type of gastrointestinal complication, and population age group.

Finally, subgroup-specific sensitivity analyses were conducted in each meta-analysis, along with a leave-one-out meta-analysis, an overall estimate of the proportion using a predefined I^2^ of 10%, and a correction for the standard error of the proportion estimate using the Knapp-Hartung method. All computational procedures were performed using Stata 18 software (StataCorp LLC, 2023, TX, USA).

### 2.11. Publication Bias

To objectively assess small-study effects, Egger’s regression asymmetry test and a funnel plot were generated.

### 2.12. Certainty of Evidence Assessment

This assessment was not performed because no specific instrument exists to evaluate the certainty of evidence in systematic reviews of proportions.

## 3. Results

**Figure 1:**
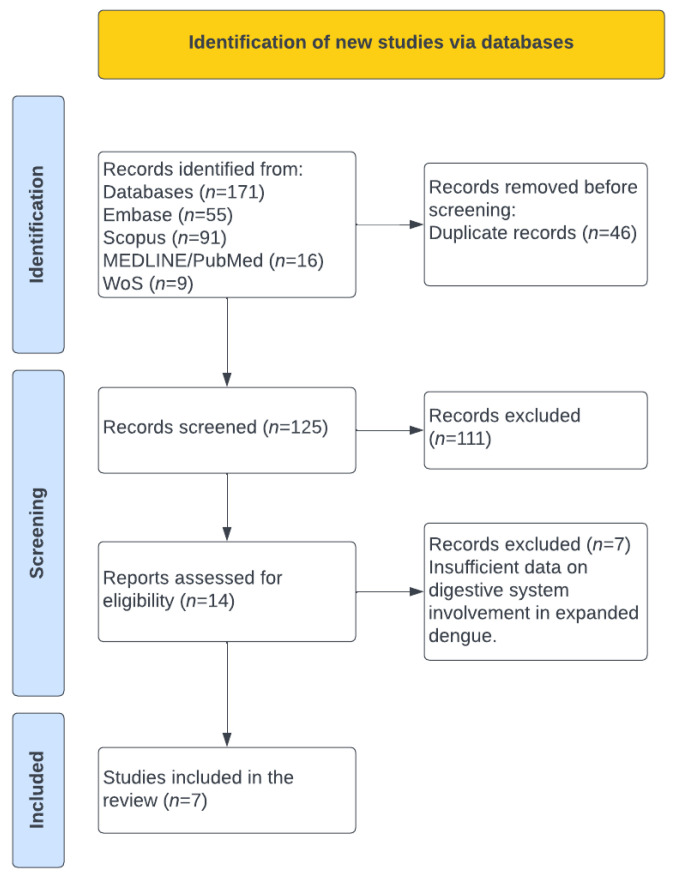
PRISMA Flow diagram for study selection.

The electronic search identified 171 articles: 16 from PubMed, 91 from Scopus, 55 from Embase, and 9 from Web of Science. After screening, 46 duplicates were removed. A total of 125 articles were assessed by title and abstract, resulting in 14 full-text articles evaluated for eligibility. Of these, 7 articles were excluded because they did not report sufficient or analyzable data to allow for an evaluation of the frequency of digestive system involvement in expanded dengue. Finally, 7 primary studies including a total of 1774 participants were included [11,12,13,14,15,16,17] (Figure 1).

### 3.1. Study Characteristics

The included articles were published between 2016 and 2024. Each study was conducted in different countries, including India [11,12,13,14], Pakistan [15,16] and Bangladesh [17], reflecting the diversity of contexts in which this issue has been investigated and enriching the global understanding of the findings (Table 1). 

Average 32. 58% of the participants were women, with ages ranging from 8 to 57 years (Table 1).

Of the included studies, 57.1% were cross-sectional [12,14,16,17] and 42.9% were retrospective cohort studies [11,13,15]. All studies (100%) were conducted in outpatient settings (Table 1).

The distribution of gastrointestinal pathologies associated with expanded dengue syndrome, stratified by organ involvement, is presented in Table 2. In addition, Table 2 reports two cases of syndromic clustering of gastrointestinal manifestations, namely the coexistence of acute acalculous cholecystitis with pancreatitis and acute acalculous cholecystitis with hepatitis [17].

Regarding the diagnostic tools used for dengue confirmation, five of the seven included studies (71.4%) employed NS1 antigen detection and/or IgM serology [11,12,13,14,17]. One study (14.3%) utilized a combination of RT-PCR, ELISA, and NS1 antigen and/or IgM testing [15], while the remaining study (14.3%) relied on ELISA and NS1 antigen and/or IgM assays [16] (Table 2).

### 3.2. Risk of Bias

**Figure 2:**
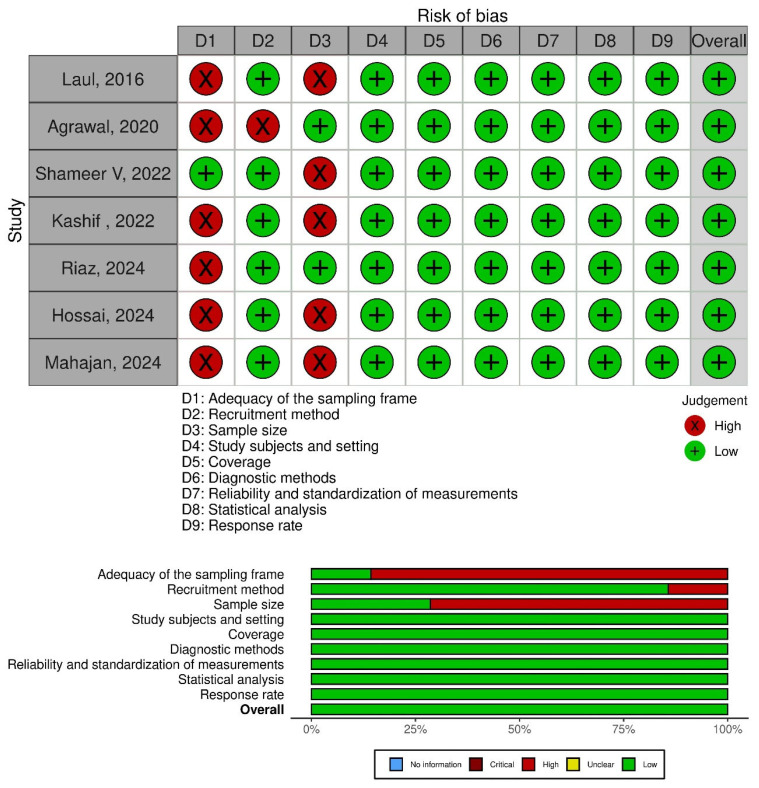
Risk of bias of the articles included [11,12,13,14,15,16,17].

Figure 2 shows the overall risk of bias assessment for the included studies. Overall, 100% of the studies were classified as having a low risk of bias, with no studies identified as having a moderate or high risk.

In the domain-specific risk of bias assessment, the highest proportion of high-risk judgments corresponded to the adequacy of the sampling frame and sample size, with deficiencies observed across all studies for the former and in several studies for the latter. These findings are mainly related to inadequate sampling frames and the lack of justification for sample size calculations. In contrast, most studies presented a low risk of bias in the domains of diagnostic method, statistical analysis, coverage, and measurement reliability, which reinforces the validity of their results.

### 3.3. Frequency of Gastrointestinal Manifestations in Expanded Dengue Syndrome

The results of the meta-analyses were classified as acute pancreatitis, acute acalculous cholecystitis, hepatitis, and fulminant hepatic failure. Other gastrointestinal alterations, including appendicitis and combined gastrointestinal presentations, were not subjected to meta-analysis, as they were reported in only one study or lacked sufficient data for quantitative synthesis (Table 1 and Table 2)

**Figure 3:**
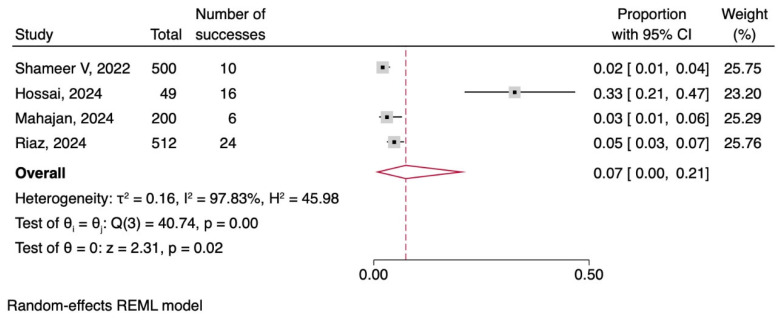
Forest plot meta-analysis of hepatic alterations in patients with dengue [11,14,15,17].

Liver Of the seven included articles, four addressed hepatic alterations in patients with dengue. The pooled frequency of hepatic involvement among patients with dengue was 7% (95% CI: 0–21), with high heterogeneity (I2 = 97.83%, *p* = 0.02), as shown in Figure 3.

Within the spectrum of hepatic pathologies, a subgroup analysis of specific diagnostic entities was performed. Among the four studies addressing hepatic involvement, three corresponded to fulminant hepatic failure and one to hepatitis. Subgroup meta-analysis revealed a pooled frequency of fulminant hepatic failure of 3% (95% CI: 2–5), whereas hepatitis accounted for 33% (95% CI: 20–47). Statistically significant differences were found between the two groups (Qb = 32.67, *p* < 0.00) suggesting that the frequency of symptoms varied significantly depending on the specific diagnosis of the hepatic alteration (Supplementary File S2).

The sensitivity analysis utilizing the leave-one-out meta-analysis demonstrated the robustness of the pooled result for the proportion of hepatic alterations. The sequential exclusion of each individual study did not significantly alter the overall pooled estimate. Although the proportions varied slightly, ranging from 3% to 10% after the omission of a study, all resulting estimations remained consistent and proximal to the general outcome (Supplementary File S3).

**Figure 4:**
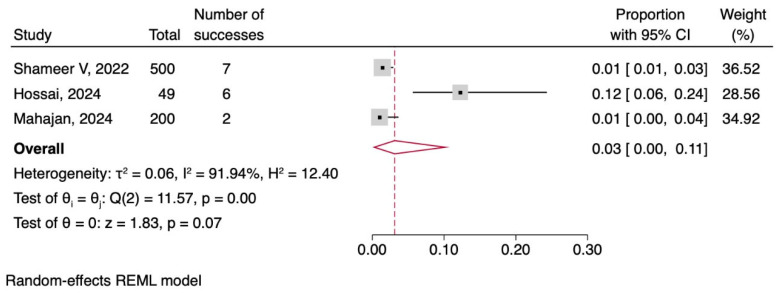
Forest plot meta-analysis of acute pancreatitis in patients with dengue [11,14,17].

Pancreas Of the 7 included articles, 3 were related to acute pancreatitis in patients with dengue. The pooled frequency of acute pancreatitis among patients with dengue was 3% (95% CI: 0–11), with heterogeneity (I2 = 91.94%, *p* = 0.07). It suggests a high of heterogeneity; however, it is of limited validity in meta-analyses with few studies (Figure 4).

The sensitivity analysis utilizing the leave-one-out meta-analysis demonstrated the robustness of the pooled result for the proportion of acute pancreatitis. The sequential exclusion of each individual study did not significantly alter the overall pooled estimate. Although the proportions varied slightly, ranging from 1% to 5% after the omission of a study, all resulting estimations remained consistent and proximal to the general outcome (Supplementary File S4).

**Figure 5:**
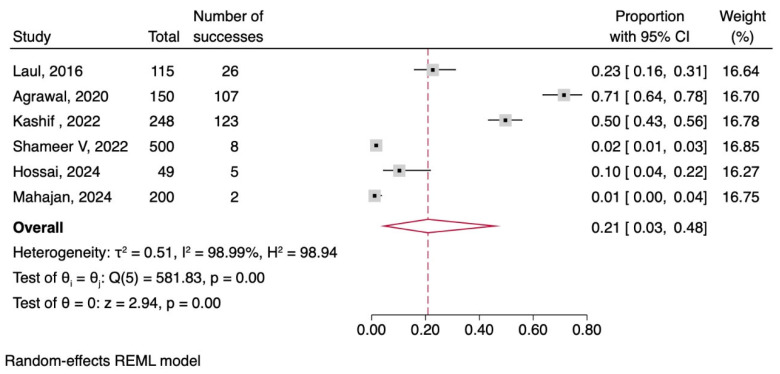
Forest plot meta-analysis of acalculous cholecystitis in patients with dengue [11,12,13,14,16,17].

Gallbladder Of the 7 included articles, 6 were related to acalculous cholecystitis in patients with dengue. The pooled frequency of acalculous cholecystitis among patients with dengue was 21% (95% CI: 3–48), with high heterogeneity (I2 = 98.99%, *p* = 0.00), as shown in Figure 5.

The sensitivity analysis utilizing the leave-one-out meta-analysis demonstrated the robustness of the pooled result for the proportion of acalculous cholecystitis. The sequential exclusion of each individual study did not significantly alter the overall pooled estimate. Although the proportions varied slightly, ranging from 16% to 27% after the omission of a study, all resulting estimations remained consistent and proximal to the general outcome (Supplementary File S5).

### 3.4. Sensitivity Analyses

The I^2^ = 10% analyses did not show relevant differences in the overall estimate. The frequencies of hepatic, pancreatic, and gallbladder involvement were 4% (95% CI: 3–5), 1% (95% CI: 1–3), and 16% (95% CI: 14–18), respectively.

The correction of the overall estimate using the Knapp-Hartung formula did not show significant differences. The corrected frequencies of hepatic, pancreatic, and gallbladder involvement were 7% (95% CI: 0–34), 3% (95% CI: 0–26), and 21% (95% CI: 1–57), respectively.

### 3.5. Source of Heterogeneity

The univariate meta-regression analyses were non-significant for the majority of the covariates analyzed in the hepatic, pancreatic, and gallbladder involvement meta-analyses. However, in the hepatic meta-analysis, the type of gastrointestinal complication was significantly associated with the reported frequency and accounted for a large proportion of between-study heterogeneity (R^2^ = 97.3%, *p* < 0.001). Nevertheless, moderate residual heterogeneity persisted (residual I^2^ = 62.65%). In the gallbladder meta-analysis, the sample size accounted for a large proportion of between-study heterogeneity (R^2^ = 80.29%, *p* < 0.001), although substantial residual heterogeneity remained (I^2^ = 94.57%).

### 3.6. Assessment of Publication Bias

The evaluation of publication bias was not conducted because fewer than ten studies were included in the analyses. According to established methodological recommendations, Egger’s regression test should not be applied when the number of studies is below this threshold, as the test has limited statistical power and may yield unreliable or spurious results under such conditions [18].

## 4. Discussion

Dengue fever (DF) is defined by the World Health Organization (WHO) as an acute febrile illness lasting 2–7 days, accompanied by at least two of the following: headache, retro-orbital pain, myalgia, arthralgia, rash, hemorrhagic manifestations, or leukopenia, in a patient from or traveling to an endemic area. According to the WHO classification, dengue is categorized into dengue without warning signs, dengue with warning signs, and severe dengue. Patients with warning signs including abdominal pain, persistent vomiting, fluid accumulation, mucosal bleeding, lethargy, hepatomegaly, rising hematocrit, and thrombocytopenia are at increased risk of progressing to severe dengue, which is characterized by significant plasma leakage leading to shock, severe bleeding, or severe organ involvement [19].

Expanded dengue refers to the occurrence of unusual or atypical organ involvement beyond the classical clinical spectrum of dengue, including neurological, hepatic, renal, or other isolated organ manifestations. Such unusual manifestations involving organs like the liver, kidneys, brain, or heart have been increasingly reported not only in dengue hemorrhagic fever (DHF) but also in dengue patients without evidence of plasma leakage. These atypical presentations may arise during different phases of the illness and can be associated with severe disease, shock, underlying host conditions, or coinfections, highlighting the broad clinical spectrum of expanded dengue [1,2,19,20].

Although there are multiple reports suggesting that expanded dengue syndrome may be associated with non-hepatic complications such as pancreatitis, cholecystitis, and appendicitis, these complications have been poorly studied in detail [6]. In this context, our study found a frequency of hepatic alterations of 7%, encompassing fulminant hepatic failure and hepatitis, as well as pancreatitis in 3% and acute acalculous cholecystitis in 21% of patients with dengue.

The association between dengue virus infection and acute pancreatitis, although documented, remains poorly understood in terms of its pathophysiology. Several theories have been proposed, including direct viral invasion of pancreatic acinar cells leading to apoptosis and necrosis, as well as viral-induced enzymatic activation resulting in immune-mediated inflammatory damage [21,22]. Additionally, microvascular ischemia due to increased vascular permeability has been suggested as a contributing factor to pancreatic injury. Moreover, DENV infection appears to induce the production of proinflammatory cytokines such as tumor necrosis factor-alpha, transforming growth factor-beta, and interleukin-10, which are also observed in cases of non-DENV-associated acute pancreatitis. Although the virus’s ability to directly infect pancreatic parenchymal cells remains unproven, it is postulated that cytokine-driven inflammation and other immune mediators may play a significant role in the extensive interstitial damage to the gland [23,24,25].

In classic dengue, the elevation of transaminases is considered an expected and “normal” finding in the course of the disease due to the virus’s tropism for the liver. Pathophysiologically, the virus invades hepatocytes and Kupffer cells to replicate, generating mild oxidative stress and transient inflammation that disrupts cellular membrane permeability. At this stage, the damage is limited, and the liver retains its regenerative capacity and function without compromising the overall health of the patient [26,27].

However, in expanded dengue, the clinical picture may progress to severe hepatitis or fulminant liver failure. Dengue hepatitis is characterized by a marked elevation of transaminases, particularly aspartate aminotransferase (AST) and alanine aminotransferase (ALT), often exceeding 1000 U/L, reflecting extensive hepatocellular injury and massive hepatic necrosis. Fulminant liver failure occurs when the liver loses its vital functions, manifesting as hepatic encephalopathy and an inability to produce coagulation factors (measured by an INR > 1.5), which can lead to death if not treated promptly in intensive care. This collapse is explained by three critical mechanisms: direct cytopathic damage, an uncontrolled immune response or “cytokine storm” inducing apoptosis, and ischemia due to hypoperfusion, caused by massive capillary leakage in severe dengue, which impairs liver oxygenation and leads to ischemic necrosis [28,29].

The pathogenesis of acute acalculous cholecystitis is considered multifactorial, involving systemic inflammatory responses, endotoxemia, cholestasis, secondary bacterial translocation, spasms of the ampulla of Vater, microangiopathic alterations, and ischemia–reperfusion injury [30,31].

Within this framework, dengue-associated acalculous cholecystitis reflects a complex immunopathological process in which a critical increase in vascular permeability represents the central mechanism. An exacerbated immune response, characterized by massive release of proinflammatory cytokines and activation of the complement system, leads to plasma extravasation, resulting in interstitial edema and marked gallbladder wall thickening. These effects are further compounded by direct viral involvement of biliary tissues, secondary cholestasis, and increased bile viscosity, collectively amplifying local inflammation and contributing to hepatobiliary involvement within the spectrum of expanded dengue syndrome [32,33].

In our study, acute acalculous cholecystitis was the most frequent gastrointestinal manifestation within expanded dengue syndrome, and this is believed to occur because the gallbladder has terminal vascularization and a serosal layer highly sensitive to changes in capillary permeability. This makes it a sentinel organ in which the cytokine storm and complement activation rapidly increase hydrostatic pressure, leading to gallbladder wall edema before other tissues are affected.

In the context of this study, a single case of appendicitis associated with dengue virus infection was identified. It is important to clarify that this finding does not represent an isolated incidence in the global literature but rather reflects the limitations inherent to the inclusion criteria of our systematic review, which excluded case reports where this association has been previously documented [34,35]. Given its unusual nature in our sample, the presence of appendicitis can be interpreted under two perspectives: as an etiological coincidence mediated by a fecalith or as a direct causality of dengue, explained by two pathophysiological mechanisms [36,37,38].

On one hand, the systemic immune response to the virus may trigger massive hyperplasia of lymphoid tissue (Peyer’s patches), obstructing the organ’s lumen and causing true obstructive appendicitis. On the other hand, a phenomenon of serositis and wall edema, analogous to the pathophysiology of acalculous cholecystitis, where plasma extravasation and fluid accumulation lead to edema of the appendiceal wall and peritoneum, may mimic or precipitate the acute inflammatory picture [39].

The presence of expanded dengue syndrome and its manifestation in gastrointestinal pathologies is directly related to the highly inflammatory nature of dengue virus infection, in which host-mediated immune responses predominate over direct cytopathic damage. In severe dengue, high levels of pro-inflammatory cytokines drive increased vascular permeability and capillary leakage, leading to systemic tissue injury rather than localized viral cytotoxicity. Research, including ongoing investigations into gut leak and microbiome contributions to severe dengue pathogenesis, supports the concept that intestinal barrier dysfunction and host inflammatory pathways play a role in disease progression to the critical phase and shock [23,31,32,33,40].

This systematic review and meta-analysis demonstrates the clinically relevant association between dengue infection and acute gastrointestinal pathologies within the spectrum of expanded dengue syndrome. Although these manifestations might be expected to occur as syndromic entities given their shared inflammatory pathophysiology, only two combined presentations acute acalculous cholecystitis with hepatitis and acute acalculous cholecystitis with pancreatitis were identified, contrasting with the anticipated pattern. This finding suggests that gastrointestinal involvement in expanded dengue syndrome more commonly manifests in an isolated rather than syndromic manner, potentially influenced by viral factors such as dengue serotype, disease severity, and timing within the clinical course, in addition to patient-related characteristics including age, sex, and underlying comorbidities.

As previously explained in the pathophysiology of dengue, the presence of gastrointestinal manifestations within the spectrum of expanded dengue syndrome (EDS) should not be considered a direct association with the critical state of the disease. If this were the case, we would expect multiorgan lesions to occur synchronously or simultaneously as part of a multiorgan failure process. However, the findings show that gastrointestinal manifestations typically appear in isolation, as evidenced by cases of acalculous cholecystitis with hepatitis or acalculous cholecystitis with pancreatitis, which were the only patterns identified. This behavior suggests that, in the context of EDS, gastrointestinal involvement does not follow the classic pattern of synchronous multiorgan failure but rather presents independently.

A direct association between dengue severity and gastrointestinal manifestations could not be evaluated, nor could the relationship between the timing or phase of the disease and the development of gastrointestinal expanded dengue manifestations, as these variables were not reported in the included studies, preventing their extraction and subsequent analysis. However, evidence from individual cohorts suggests a possible link between disease progression, severity, and atypical involvement. In a pediatric cohort, atypical manifestations were more frequently observed in patients who progressed to severe dengue. Clinically, warning signs such as persistent abdominal pain, prolonged shock, and lack of improvement after defervescence were described, indicating that atypical manifestations tended to emerge as the disease advanced toward the critical phase. Moreover, the proportion of atypical presentations was higher among children classified with severe dengue compared to non-severe cases, supporting a potential association between temporal progression, increasing clinical severity, and the development of expanded organ involvement [41].

In this regard, epidemiological evidence indicates that DENV-2 has been identified as a risk factor for severe dengue in both Asia and the Americas, despite exhibiting the lowest seroprevalence in the latter region, while DENV-3 shows an inconsistent association with severe disease, including a negative association in Asia and no significant association in the Americas [42]. Furthermore, specific genetic changes in DENV-2 and DENV-3 have been linked to outbreaks with increased clinical severity in regions such as the Americas and India [42,43].

In tropical settings where expanded dengue syndrome with a predominant gastrointestinal phenotype is observed, these manifestations are considered part of the severe dengue spectrum and appear to be favored by more virulent serotypes or genotypes, as well as by secondary infections [44].

Nevertheless, no specific viral variant has been conclusively shown to directly drive an increased incidence of gastrointestinal manifestations. Instead, gastrointestinal involvement likely results from a complex interaction between circulating viral variants, pre-existing host immunity, and additional host-related factors. Importantly, all dengue virus serotypes (DENV-1 to DENV-4) have the potential to cause severe dengue and expanded dengue syndrome with gastrointestinal involvement, including hepatitis, cholecystitis, pancreatitis, gastrointestinal bleeding, and ascites [23,24,32,44,45].

Despite the biological plausibility of these associations, they could not be systematically assessed in the present analysis, as the included studies did not consistently or precisely report serotype, genotype, immune status, or clinical timing in relation to gastrointestinal involvement, representing a key limitation and an important avenue for future research.

From a clinical standpoint, recognizing these isolated gastrointestinal manifestations as part of expanded dengue syndrome is essential, as their early identification may avert unnecessary surgical interventions and serve as an early indicator of progression toward the critical phase of the disease.

## 5. Conclusions

Clinically relevant gastrointestinal involvement is a significant component of expanded dengue syndrome, with acute acalculous cholecystitis as the most frequent manifestation, followed by hepatic and pancreatic complications. Symptoms described within the spectrum of expanded dengue have been more prevalently reported in endemic dengue areas and may reflect a more severe or complicated clinical course. Early recognition of these atypical digestive presentations may help prevent unnecessary surgical interventions and improve clinical outcomes.

## Figures and Tables

**Table 1:** Characteristics of the included studies.

Author, Year	Country	N° Patients/N° Cases n (%)	Age (Years)	Males n (%)	Study Design
Laul, 2016 [12]	India	115/26 (22.6%)	31.36 ± 13.17 *	64 (55.7%)	Cross-sectional study
Agrawal, 2020 [13]	India	150/107 (71.3%)	35.03 ± 13.75 *	107 (71.3%)	Retrospective cohort study
Shameer, 2022 [14]	India	500/26 (5.2%)	42 ± 15 *	326 (65.2%)	Cross-sectional study
Kashif, 2022 [16]	Pakistan	248/123 (49.6%)	28.7 ± 11.1 *	134 (54.0%)	Cross-sectional study
Mahajan, 2024 [11]	India	200/10 (5.0%)	12 (8–15) **	178 (89.0%)	Retrospective cohort study
Hossain, 2024 [17]	Bangladesh	49/25 (51.0%)	33 ± 9 *	31 (63.3%)	Cross-sectional study
Riaz, 2024 [15]	Pakistan	512/24 (4.7%)	34.1 ± 15.1 *	356 (69.5%)	Retrospective cohort study

* Average ± Standard deviation; ** Median (Minimum–Maximum).

**Table 2:** Digestive system manifestations of expanded dengue.

**LIVER**					
**Study**	**Disease**	**Population**	**Cases**	**Age Group**	**Diagnostic Tool for** **Dengue**
Shameer, 2022 [14]	Fulminant hepatic failure	500	10	Pediatric and Adults	NS1 or/and IgM
Mahajan, 2024 [11]	Fulminant hepatic failure	200	6	Pediatric	NS1 or/and IgM
Hossain, 2024 [17]	Hepatitis	49	16	Adults	NS1 or/and IgM
Riaz, 2024 [15]	Fulminant hepatic failure	512	24	Adults	RT-PCR/ELIZA/NS1 or/and IgM
**PANCREAS**					
**Study**	**Disease**	**Population**	**Cases**	**Age** **Group**	**Diagnostic Tool for** **Dengue**
Shameer, 2022 [14]	Acute pancreatitis	500	7	Pediatric and Adults	NS1 or/and IgM
Mahajan, 2024 [11]	Acute pancreatitis	200	2	Pediatric	NS1 or/and IgM
Hossain, 2024 [17]	Acute pancreatitis	49	6	Adults	NS1 or/and IgM
**GALLBLADDER**					
**Study**	**Disease**	**Population**	**Cases**	**Age** **Group**	**Diagnostic Tool for** **Dengue**
Laul, 2016 [12]	Acalculous cholecystitis	115	26	Adults	NS1 or/and IgM
Agrawal, 2020 [13]	Acalculous cholecystitis	150	107	Adults	NS1 or/and IgM
Shameer, 2022 [14]	Acalculous cholecystitis	500	8	Pediatric and Adults	NS1 or/and IgM
Kashif, 2022 [16]	Acalculous cholecystitis	248	123	Adults	ELIZA/NS1 or/and IgM
Mahajan, 2024 [11]	Acalculous cholecystitis	200	2	Pediatric	NS1 or/and IgM
Hossain, 2024 [17]	Acalculous cholecystitis	49	5	Adults	NS1 or/and IgM
**INTESTINE**					
**Study**	**Disease**	**Population**	**Cases**	**Age** **Group**	**Diagnostic Tool for** **Dengue**
Shameer, 2022 [14]	Appendicitis	500	1	Pediatric and Adults	NS1 or/and IgM
**GASTROINTESTINAL SYNDROME**					
**Study**	**Syndrome**	**Population**	**Cases**	**Age** **group**	**Diagnostic Tool for** **Dengue**
Hossain, 2024 [17]	Acalculous cholecystitis And Acute pancreatitis	49	1	Adults	NS1 or/and IgM
Hossain, 2024 [17]	Acalculous cholecystitis And Hepatitis	49	1	Adults	NS1 or/and IgM

## Data Availability

The original contributions presented in this study are included in the article/Supplementary Material. Further inquiries can be directed to the corresponding author.

## Supplementary Materials

The following supporting information can be downloaded at: https://www.mdpi.com/article/10.3390/tropicalmed11030077/s1, Supplementary File S1. Complete search strategy; Supplementary File S2. Forest plot of hepatic alterations: subgroup analysis by type of hepatic condition. Supplementary File S3. Leave-one-out meta-analysis for the frequency of hepatic alterations. Supplementary File S4. Leave-one-out meta-analysis for the frequency of acute pancreatitis. Supplementary File S5. Leave-one-out meta-analysis for the frequency of acalculous cholecystitis.
