# Supplementary material for: Expanded Dengue and the Digestive System: A Systematic Review and Meta-Analysis
Source: Trop Med Infect Dis. 2026 Mar 7;11(3):77. doi: 10.3390/tropicalmed11030077 (PMC13029969; doi:10.3390/tropicalmed11030077)
Supplement: Supplementary file 1 [file tropicalmed-11-00077-s001.zip › Supplementary File S1. Complete search strategy_20260226.pdf]

PUBMED

1) "Dengue"[Mesh] AND "Gastrointestinal Diseases"[Mesh] 79 Result
2) ("expanded dengue"[All Fields] OR "atypical dengue"[All Fields]) 79 results
3) ("Gastrointestinal Diseases"[Majr] OR "Digestive System Diseases"[Mesh] OR "gastrointestinal manifestations"[All Fields] OR "gastrointestinal involvement"[All Fields] OR "gastrointestinal complications"[All Fields] OR "Esophageal Diseases"[Majr] OR "esophageal disease"[All Fields] OR "esophageal disorders"[All Fields] OR "Stomach Diseases"[Majr] OR "gastric disease"[All Fields] OR "gastric disorders"[All Fields] OR "Intestinal Diseases"[Majr] OR "small intestinal disease"[All Fields] OR "enteritis"[All Fields] OR "Colonic Diseases"[Majr] OR "colon disease"[All Fields] OR "colitis"[All Fields] OR "Liver Diseases"[Majr] OR "hepatic disease"[All Fields] OR "hepatic disorders"[All Fields] OR "hepatitis"[All Fields] OR "Biliary Tract Diseases"[Majr] OR "gallbladder disease"[All Fields] OR "biliary tract disorders"[All Fields] OR "Pancreatic Diseases"[Majr] OR "pancreatic disease"[All Fields] OR "pancreatic disorders"[All Fields] OR "pancreatitis"[All Fields]) 2,247,542 results
4) #2 AND #3 16 results

BIBLIOTECA VIRTUAL DE SALUD

1) ("dengue expandido" OR "dengue atípico") 2 result
2) (mh:"Enfermedades Gastrointestinales" OR mh:"Enfermedades del Sistema Digestivo" OR "manifestaciones gastrointestinales" OR "compromiso gastrointestinal" OR "complicaciones gastrointestinales" OR mh:"Enfermedades del Esófago" OR "enfermedad esofágica" OR "trastornos esofágicos" OR mh:"Enfermedades del Estómago" OR "enfermedad gástrica" OR "trastornos gástricos" OR mh:"Enfermedades Intestinales" OR "enfermedad del intestino delgado" OR enteritis OR mh:"Enfermedades del Colon" OR "enfermedad del colon" OR colitis OR mh:"Enfermedades del Hígado" OR "enfermedad hepática" OR "trastornos hepáticos" OR hepatitis OR mh:"Enfermedades de las Vías Biliares" OR "enfermedad de la vesícula biliar" OR "trastornos de la vía biliar" OR mh:"Enfermedades del Páncreas" OR "enfermedad pancreática" OR "trastornos pancreáticos" OR pancreatitis) 42 238 result
3) #2 AND #3 0

SCIELO

1) ("expanded dengue" OR "atypical dengue" OR "dengue expandido" OR "dengue atípico") 0 result
2) ("gastrointestinal diseases" OR "digestive system diseases" OR "gastrointestinal manifestations" OR "gastrointestinal involvement" OR "gastrointestinal complications" OR "enfermedades gastrointestinales" OR "enfermedades del sistema digestivo" OR "manifestaciones gastrointestinales" OR "compromiso gastrointestinal" OR "complicaciones gastrointestinales" OR "esophageal disease" OR "esophageal disorders" OR "enfermedad esofágica" OR "trastornos esofágicos" OR "gastric disease" OR "gastric disorders" OR "enfermedad gástrica" OR "trastornos gástricos" OR "intestinal diseases" OR "small intestinal disease" OR enteritis OR "enfermedades intestinales" OR "colonic disease" OR colitis OR "enfermedades del colon" OR "liver disease" OR "hepatic disease" OR "hepatic disorders" OR hepatitis OR "enfermedades hepáticas" OR "biliary tract disease" OR "gallbladder disease" OR "biliary tract

disorders" OR "enfermedades de las vías biliares" OR "pancreatic disease" OR "pancreatic disorders" OR pancreatitis OR "enfermedades del páncreas") 8 644
3) #2 AND #3 0 result

Embase

1) ('expanded dengue':ti,ab,kw OR 'atypical dengue':ti,ab,kw) 90 result
2) ('gastrointestinal disease'/exp OR 'digestive system disease'/exp OR 'gastrointestinal manifestation':ti,ab,kw OR 'gastrointestinal involvement':ti,ab,kw OR 'gastrointestinal complication':ti,ab,kw OR 'esophageal disease'/exp OR 'esophageal disease':ti,ab,kw OR 'esophageal disorder':ti,ab,kw OR 'stomach disease'/exp OR 'gastric disease':ti,ab,kw OR 'gastric disorder':ti,ab,kw OR 'intestinal disease'/exp OR 'small intestinal disease':ti,ab,kw OR 'enteritis':ti,ab,kw OR 'colon disease'/exp OR 'colon disease':ti,ab,kw OR 'colitis':ti,ab,kw OR 'liver disease'/exp OR 'hepatic disease':ti,ab,kw OR 'hepatic disorder':ti,ab,kw OR 'hepatitis':ti,ab,kw OR 'biliary tract disease'/exp OR 'gallbladder disease':ti,ab,kw OR 'biliary tract disorder':ti,ab,kw OR 'pancreatic disease'/exp OR 'pancreatic disease':ti,ab,kw OR 'pancreatic disorder':ti,ab,kw OR 'pancreatitis':ti,ab,kw) 4,726,489 result
3) #1 AND #2 55 result

Scopus

1) TITLE-ABS-KEY("expanded dengue" OR "atypical dengue") 819 Result
2) ("gastrointestinal disease" OR "digestive system disease" OR "gastrointestinal manifestation*" OR "gastrointestinal involvement" OR "gastrointestinal complication*" OR "esophageal disease*" OR "esophageal disorder*" OR "stomach disease*" OR "gastric disease*" OR "gastric disorder*" OR "intestinal disease*" OR "small intestinal disease*" OR enteritis OR "colon disease*" OR colitis OR "liver disease*" OR "hepatic disease*" OR "hepatic disorder*" OR hepatitis OR "biliary tract disease*" OR "gallbladder disease*" OR "pancreatic disease*" OR "pancreatic disorder*" OR pancreatitis) 1,194,374 Result
3) #1 AND #2 91

Web of Science

1) TS=("expanded dengue" OR "atypical dengue") 71 Result
2) TS=("gastrointestinal disease" OR "digestive system disease" OR "gastrointestinal manifestation*" OR "gastrointestinal involvement" OR "gastrointestinal complication*" OR "esophageal disease*" OR "esophageal disorder*" OR "stomach disease*" OR "gastric disease*" OR "gastric disorder*" OR "intestinal disease*" OR "small intestinal disease*" OR enteritis OR "colon disease*" OR colitis OR "liver disease*" OR "hepatic disease*" OR "hepatic disorder*" OR hepatitis OR "biliary tract disease*" OR "gallbladder disease*" OR "pancreatic disease*" OR "pancreatic disorder*" OR pancreatitis) 715,618 Result
3) #1 AND #2 9 Result

CENTRAL - Cochrane Central Register of Controlled Trials

1) ("expanded dengue" OR "atypical dengue") 1 Result
2) ("gastrointestinal diseases" OR "digestive system diseases" OR "gastrointestinal manifestations" OR "gastrointestinal involvement" OR "gastrointestinal complications"

OR "esophageal diseases" OR "esophageal disease" OR "esophageal disorders" OR "stomach diseases" OR "gastric disease" OR "gastric disorders" OR "intestinal diseases" OR "small intestinal disease" OR "enteritis" OR "colonic diseases" OR "colon disease" OR "colitis" OR "liver diseases" OR "hepatic disease" OR "hepatic disorders" OR "hepatitis" OR "biliary tract diseases" OR "gallbladder disease" OR "biliary tract disorders" OR "pancreatic diseases" OR "pancreatic disease" OR "pancreatic disorders" OR "pancreatitis") 132 525 Results

3) #1 AND 2 0
