# Supplementary figures and images for: Expanded Dengue and the Digestive System: A Systematic Review and Meta-Analysis
Source: Trop Med Infect Dis. 2026 Mar 7;11(3):77. doi: 10.3390/tropicalmed11030077 (PMC13029969; doi:10.3390/tropicalmed11030077)

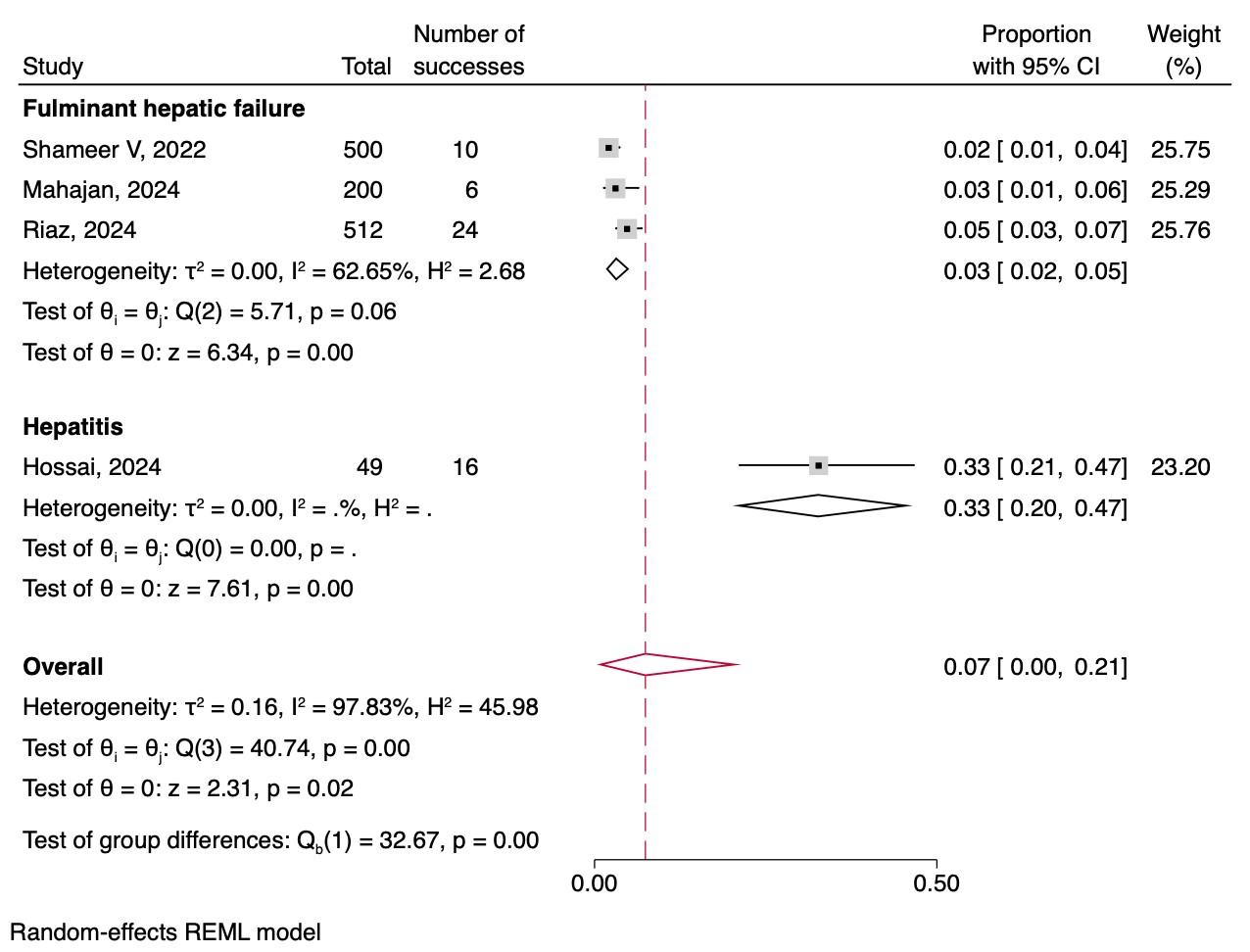

Supplement: Supplementary file 1 [file tropicalmed-11-00077-s001.zip › Supplementary File S2. Forest plot of hepatic alterations subgroup analysis by type of hepatic condition..jpg]

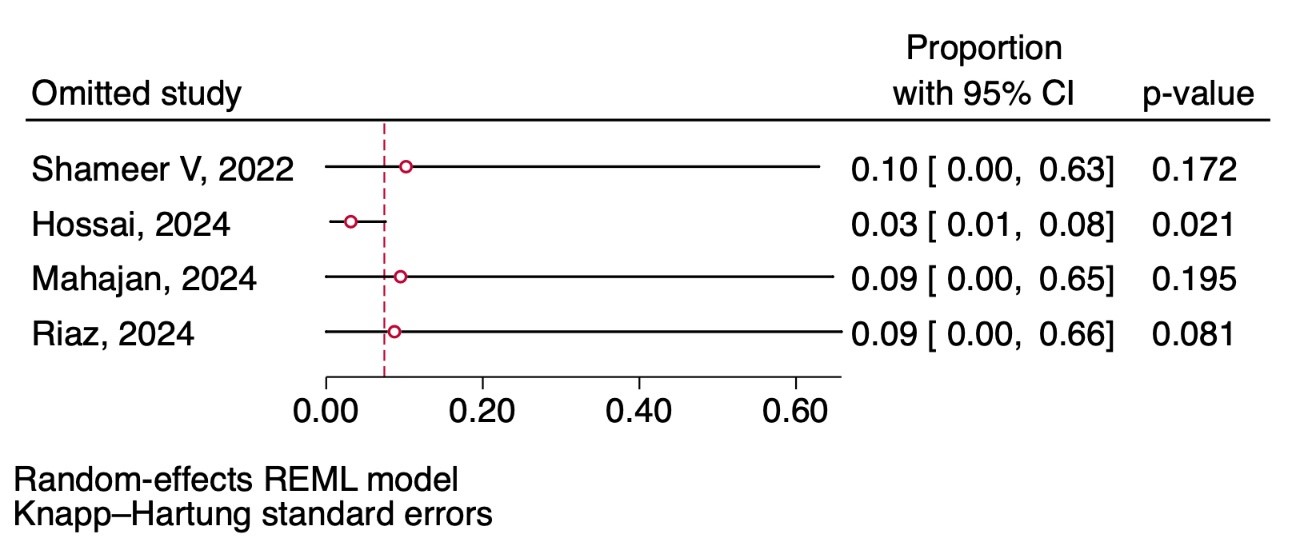

Supplement: Supplementary file 1 [file tropicalmed-11-00077-s001.zip › Supplementary File S3. Leave-one-out meta-analysis for the frequency of hepatic alterations..jpg]

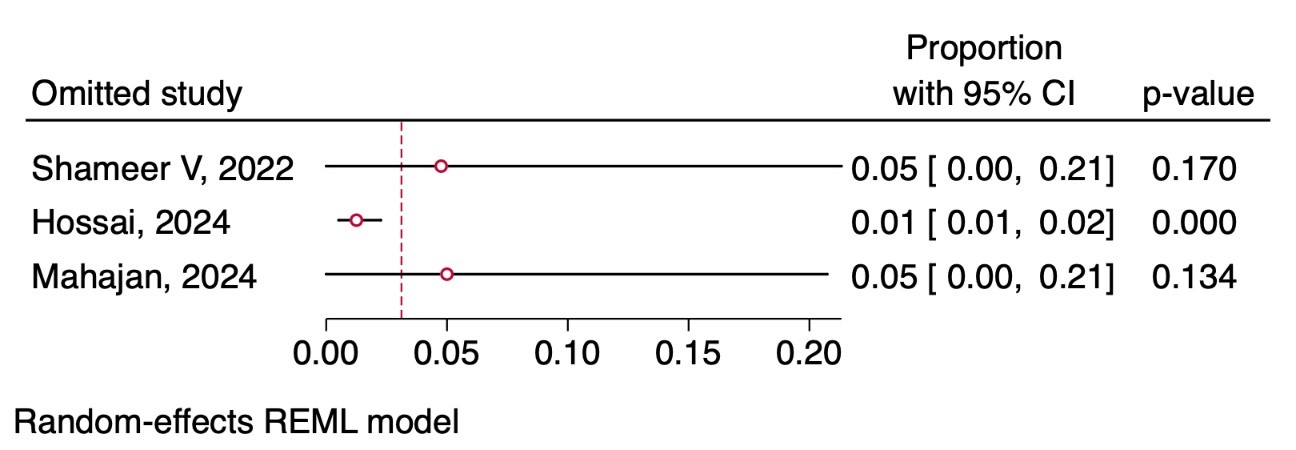

Supplement: Supplementary file 1 [file tropicalmed-11-00077-s001.zip › Supplementary File S4. Leave-one-out meta-analysis for the frequency of acute pancreatitis..jpg]

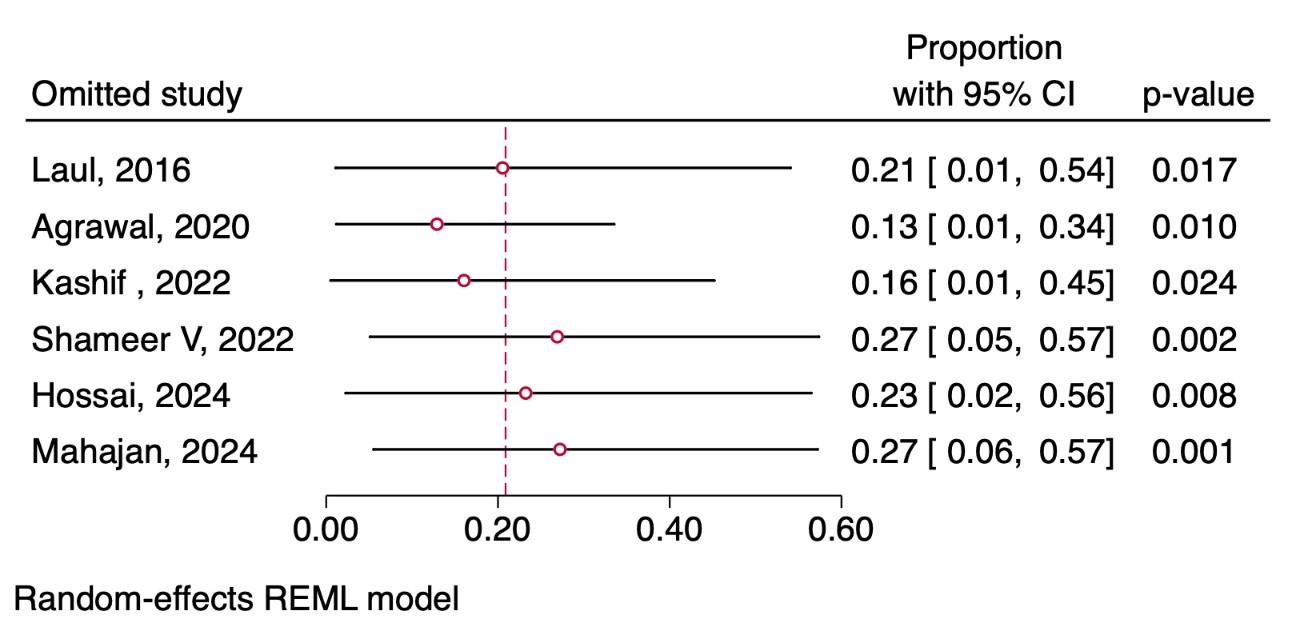

Supplement: Supplementary file 1 [file tropicalmed-11-00077-s001.zip › Supplementary File S5. Leave-one-out meta-analysis for the frequency of acalculous cholecystitis..jpg]
